# Cancer Clinical Trials in Africa—An Untapped Opportunity: Recommendations From AORTIC 2019 Conference Special Interest Group in Clinical Trials

**DOI:** 10.1200/GO.21.00096

**Published:** 2021-09-10

**Authors:** Abiola Ibraheem, Colin Pillai, Ifeoma Okoye, J. Joshua Smith, Diane Reidy-Lagunes, Grace Macaulay, Olusegun Alatise

**Affiliations:** ^1^ Section of Hematology Oncology, University of Chicago, Chicago, IL; ^2^ Division of Clinical Pharmacology, University of Cape Town, Cape Town, South Africa; ^3^ CP+ Associates GmbH, Basel, Switzerland; ^4^ Department of Radiology, College of Medicine, University of Nigeria, Nsukka, Nigeria; ^5^ University of Nigeria Centre for Clinical Trials, University of Nigeria Teaching Hospital, Enugu, Ituku Ozalla, Nigeria; ^6^ Colorectal Service, Department of Surgery, Memorial Sloan Kettering Cancer Center, New York, NY; ^7^ Gastrointestinal Oncology Service, Department of Medicine, Memorial Department of Medicine, Weill Cornell Medical College, New York, NY; ^8^ Medical Scientific Affairs and Strategy (Oncology), Cepheid Oncology, Sunnyvale, CA; ^9^ Division of Gastrointestinal/Surgical Oncology, Department of Surgery, Obafemi Awolowo University/Teaching Hospitals, Ile-Ife, Nigeria

## Abstract

Cancer is now a formidable health care burden in sub-Saharan Africa (SSA) due to lifestyle westernization and longer life expectancy. The exponential increase in cancer incidence coupled with high mortality rate is not comparable with that seen in westernized countries. To address global cancer disparity, globalization of cancer clinical trials to involve sub-Saharan Africa can serve as a platform where innovative targeted therapies can be made available to patients in the environ. In the 2019 African Organization for Research and Training in Cancer (AORTIC) conference held at Maputo, Mozambique, a group of clinical trialists spanning across multiple continents highlighted the opportunities in Africa for the conduct of cancer clinical trials. The secondary purpose of the meeting was to address the belief that Africa was incapable of conducting interventional cancer trials but showed the in-continent strengths, such as available capacities, trained local clinical trialists with clinical trial experiences, clinical trial consortia, local capabilities, mapping out logistics, ethical consideration, political will, real-time benefits of clinical trials to clinical practice, and future directions for trials.

## INTRODUCTION

There has been an increase in globalization of clinical trials in the beginning of the 21^st^ century, driven by multiple factors including the need to access wider pools of study participants, reduce research timelines, and address the global burden of disease. Over the past decades, there has been an expansion of cancer clinical trials from resource rich-countries to involve other countries with lesser resources.^1-3^ Factors cited for globalization of cancer clinical trials include the ability to reduce operational costs, access to treatment-naïve patients, growth of the health care market size, the increase of research capacity, and demands for local patient data by regulatory authorities (notably those in Asia).^4,5^ The benefits of globalizing cancer trials have resulted in increased geographic dispersion of clinical development operations, provided a lever for governments to negotiate drug prices, improved availability and experience with new therapies to patients with cancer and their attending clinicians, and improved health care systems. Sadly, most African countries (except for few countries such as Egypt and South Africa) have been left out of these globalization efforts.

**CONTEXT**

**Key Objective**
To summarize the recommendations from the African Organization for Research and Training in Cancer Conference Special interest group in Clinical Trials.
**Knowledge Generated**
We highlight the opportunities and effective approaches to conducting interventional cancer clinical trials in sub-Sharan Africa.
**Relevance**
Toward reducing global cancer disparity, the conduct of cancer clinical trials in Africa promises to make available innovative therapies to African patients.

The participation of African countries in clinical trials is arguably the most proactive route to bringing innovative cancer therapies and functional infrastructure to the continent. Some of the reasons cited for excluding Africa in clinical trials include low resources,^6^ poor infrastructure, limited expertise, concerns about data quality and integrity, and post-trial access of the trial medications to the participants and the population after the trial is completed. It is a reality, however, that these reasons have not prevented the conduct and success of infectious diseases clinical trials such as HIV and/or AIDS and tuberculosis. The insights in population-level treatment regimens for HIV/AIDS studies conducted in Africa have improved care for people living with this disease worldwide and the inclusion of Africa would be advantageous to the sponsors of clinical trials (eg, the pharmaceutical industry).^7^

To that end, the African Organization for Research and Training in Cancer (AORTIC) met in Maputo, Mozambique, in November 2019 to discuss effective approaches to conducting cancer clinical trials in Africa and the opportunities that the continent provides as a hub for cancer clinical trials. The purpose of this paper is to summarize the key presentations and to present recommendations that arose from the meeting to invite further collaboration.

## THE CURRENT STATUS OF ONCOLOGY CLINICAL TRIALS IN AFRICA

Although Africa has approximately 15% of the world's population,^8^ only an estimated 2% of global clinical trials are conducted in Africa.^9^ A review of the National Institutes of Health trial repository ClinicalTrials.gov^10^ shows that 736 clinical trials are conducted in Africa, out of which only 26 are cancer-related interventional trials and only six of these trials are conducted in countries with predominantly Black patients.

The apparent noninclusion of African patients is multifactorial and includes cultural beliefs, lack of trust, and concerns around exploiting vulnerable populations. It is interesting to note that under-representation of African patients is also seen in the global north as evidenced by advocacy efforts to diversify clinical trials and improve representation of racial and ethnic minority groups. It is important to advocate for the inclusion of African patients in cancer clinical trials because these patients are more likely to present at a later stage or have a poorer outcome.

Africa's research landscape is beginning to change because of increase in economic development, Westernized lifestyle, and the rising incidence of noncommunicable diseases such as cancer. African countries are under-represented in cancer research partly because of lack of research resources,^6^ although it is widely known that research-led solutions are impactful on the high rates of mortality.^6,11^ Organizations such as AORTIC and ASCO are building bridges to conquer cancer and reduce disparities in patient outcome through educational programs and partnership with African countries.^12^ In addition, regulatory agencies such as the US Food and Drug Administration have convened discussion meetings and issued position papers and guidance documents in an effort to address this disparity.^13^

## GOOD CLINICAL PRACTICE, ETHICS, AND REGULATORY ISSUES IN AFRICA

Several individual regulatory bodies have joined regional harmonization efforts to ensure that the conduct of clinical trials in African countries adheres with international good clinical practice and ethical standards. For example, the Pan African Clinical Trials Registry has built a platform to register trials and improve transparency.^14,15^ The African Vaccines Regulatory Forum (AVAREF), established in 2006, is a pan-African network that provides clinical trial oversight and other capacity development activities to increase access to safe and effective medical products. AVAREF improves collaborative networks and strategic alliances by promoting efficient clinical trial platforms, shared best practices, and templates for clinical trial assessment procedures. The African Medicine Agency serves as the continental regulatory body that ensures harmonized and strengthened regulatory systems by governing the regulation of medicines and medical products in Africa.^16^

In Nigeria, to address Good Clinical Practice, The Association for Good Clinical Practice in Nigeria in collaboration with the National Agency for Food and Drug Administration and Control aimed at increasing the participation of African indigenous scientists in global clinical trials to build capacity. Regulatory Resources For Africa is a collaboration among regulatory bodies in 34 African countries for clinical trial application and harmonization.^17^ Although some agencies have internal capacity for scientific and ethical review, in most cases where there is no capacity for review, assessments are outsourced to academia.

The adherence to the international guidelines on ethical conduct will protect vulnerable populations and encourage sponsors to study, register, market, and ensure affordable access to state-of-the-art new therapies in African countries.

## DESIGN, CONDUCT, AND ANALYSIS OF PHASE I-IV CLINICAL TRIALS IN AFRICA

Phase I trials representing the important translation from preclinical to human use establishes safety, pharmacokinetics, and early assessment of dose response. These have been classically used for both cancer and noncancer drugs in diverse global settings.^18^ In Africa, historically, there are very few phase I studies, but this is changing with some phase I infectious disease interventional clinical trials reported for antimalarial drugs,^19^ Ebola vaccines,^20^ and HIV/AIDS vaccines.^21^ Although very few phase I cancer clinical trials are reported in Africa (eg, colon cancer phase I studies),^22,23^ the fact that Phase I trials have been conducted in Africa indicates that the scientific skills and infrastructure already exists and can be expanded in future.

Phase II trials demonstrate further evidence of safety and efficacy in patients before moving to larger phase III trials that evaluate the efficacy of a drug based on recognized clinical end points. For example, in Mali, a single-blind, randomized primaquine study in 80 patients used infectivity before and after treatment as the primary efficacy end point.^24^ There are few ongoing phase II oncology trials in Africa, eg, an investigator-initiated Phase II breast cancer clinical trial in Nigeria where the response rate of subcutaneous trastuzumab is being assessed.^25^ This lends evidence that Phase II trials can be accomplished in Africa.

Phase III studies confirm therapeutic efficacy observed in phase II trials in a larger number of patients and assess additional clinical questions (eg, comorbidities to monitor during treatment, dosage adjustment, length of treatment, profiles of responder patients, and drug interactions). Phase III studies require large sample sizes and long study durations and are therefore more expensive. Some have argued that Africa provides a low-cost opportunity with large patient populations that can ensure rapid accrual with increased incidence of the disease.^26^ These trials need to align with the national health strategies and burden of disease priorities. These studies should have inclusion and exclusion criteria that are not too vague (heterogeneous) or too aggressive (not translatable) to optimally generate data that are more representative and informative of patients of African Ancestry. Strong and informed regulatory and government agencies can help facilitate this.

## DRUG DISCOVERY AND DEVELOPMENT IN AFRICA: EXPANSION THROUGH COLLABORATIONS

The people in the 54 sovereign countries in Africa are diverse in physiologic and genetic makeup, culture, and the intrinsic and extrinsic factors that ICH-E5^27^ describes, all of which could be used positively in clinical drug development to benefit global patients. Drug development on endemic infectious diseases such as malaria, tuberculosis, and HIV/AIDS illustrates the profound impact that drug development could have in Africa. The lessons learnt from infectious disease drug development could be the platform to increase oncology drug development and participation in global oncology trials. As an illustrative example of the missed opportunity, a search of ClinicalTrials.gov showed that out of the over 1,000 clinical trials using pembrolizumab in all cancers, 16 were in Africa; as mentioned above, the majority of these were in South Africa (15) and Egypt (one).^28^

Africa's research capabilities and infrastructure are expanding through collaborations with international organizations such as The European and Developing Countries Clinical Trials Partnership, which was created in 2003 to develop capabilities for phase II or III trials in sub-Saharan Africa. The Swiss Tropical and Public Health Institute has developed TRREE,^29^ a web site that provides ethical guidelines, training, and access to free distance learning programs and resources to support studies conducted in Africa.

The readiness of Africa has been facilitated through the capacity-building investments mentioned above and additionally in programs such as Welcome Centre for Anti-Infectives Research and the Holistic Drug Discovery and Development Centre. The Holistic Drug Discovery and Development Centre put its first African drug MMV390048 through phase I and II clinical trials as a promising agent working on all stages of the malaria parasite's life cycle.^30,31^

Capacity building in Africa has been extended beyond drug development to molecular diagnostics in infectious diseases, for example, using Cepheid's platform to improve the diagnosis of HIV and tuberculosis.^32^ This is being extended gradually into oncology with partnerships between nongovernmental organizations such as the Max Foundation and pharmaceutical companies that are providing imatinib for the treatment of chronic myelogenous leukemia with monitoring of the patient using the BCR-ABL GeneXpert Ultra.^33^

## CONDUCTING CLINICAL TRIALS IN AFRICA AND THE OPPORTUNITIES TO SCALE UP

To scale up oncology clinical trials in Africa, we have identified seven strategic areas that must evolve in many countries to correct the current low number of cancer clinical trials in Africa.

1. Development of human capacity in conducting clinical trials:

In 2019, to address the extreme limitation in human capacity, The African Research Group for Oncology (ARGO),^34^ supported by the US Civilian Research and Development Foundation, funded and organized a training program on clinical trials in Nigeria for 50 early career researchers. Pre- and post-training assessment revealed a significant rise in knowledge. The African Clinical Trials Consortium^35^ organizes monthly webinars on different aspects of clinical trials with the aim of building a critical mass of clinical trialists in Africa.^35^ These initiatives will develop the confidence of clinical trial sponsors and funding agencies to invest further in the continent. We encourage more of such opportunities to expose and train early career researchers by using stratified training (basic and advanced courses) and hands-on workshops. In addition to training early career researchers, it is necessary to train mentors on how to provide necessary support for these young researchers. We suggest that the AORTIC and other international organizations partner with societies and training institutions to organize capacity development training.

2. Increasing visibility of the research infrastructure for interventional cancer clinical trial in Africa:

The African Academy of Sciences recently launched the Clinical Trials Community^36^ project, a database of African clinical trial sites and capabilities as an open-access, web-based, up-to-date system with profiles of African clinical trial sites, and the associated ecosystem of regulatory and ethics committees for clinical trials on the continent. This project has impressive local and international support from private and public sector partners including the Bill & Melinda Gates Foundation, the US National Institutes of Health, the biopharmaceutical industry, product development partners, clinical researchers, and African regulatory agencies. The database is expected to enable an increase in investment in clinical trials on the continent.

The following areas have been identified as gaps that the project will seek to address.

- Up-to-date information on country-specific clinical trial regulatory guidelines is part of the platform under a collaboration with AVAREF and supported by regulatory agencies from the member states.
- Up-to-date information on key clinical trial site capabilities and gaps will be critical for sponsors to target their more detailed subsequent trial-specific feasibility assessments.
- A graphical overlay of disease burden data onto the location of existing clinical trial sites will help to simultaneously identify where trials could be located and to target capacity and infrastructure development programs
- Early prioritization of diseases and therapeutic areas of mutual interest to Africa, the African Academy of Sciences, and its partners such as tuberculosis, sickle cell disease, several oncology indications, and Lassa fever.

To better describe the clinical trial infrastructure for oncology trials, Bio Ventures for Global Health^37^ developed a complementary initiative to profile cancer clinical trial sites and describe available technologies and infrastructures in their sites. African countries and institutions have varying degrees of sophistication in their research infrastructure for interventional clinical trials. It is expected that research infrastructure should include major scientific equipment, software as well as knowledge-based resources with associated technical support.

3. Strengthen the African trials consortia:

The formation of consortiums in Africa will offer the ability to rapidly recruit many patients into a trial while also allowing for collaboration among African clinical trialists. A few such consortia are beginning to emerge, some of which have been recognized by the US National Cancer Institute, eg, ARGO^34^ working on colorectal and breast cancers; Prostate Cancer Transatlantic Consortium and Men of African Descent and Cancer of the Prostate working on prostate cancer; and the Global Surgical Outcome Collaboration working on surgical outcomes in oncology. Global Surgical Outcome Collaboration has successfully conducted and is conducting clinical trials in Africa, which include pragmatic multicenter FActorial randomized controlled triaL testing measures to reduCe surgical site infection in lOw- and middle-income couNtries (FALCON trial)^38^ and a cluster randomized trial of sterile glove and instrument change at wound closure to reduce surgical site infection (CHEETAH trial). Some international consortia involves African and European countries, e.g., one being led by the Kintampo Health Research Centre in Ghana and involving partners from the KEMRI-Wellcome Trust Research Programme in Kenya, the Centre National de Recherche et de Formation surle Paludisme, Ouagadougou in Burkina Faso, and the London School of Hygiene and Tropical Medicine in the United Kingdom. This consortium focuses on the delivery of health care to children in low-resource settings.

4. Establishment of biorepositories:

Most drug clinical trials require collection of biologic fluid and tissue samples and require careful protocols for sample processing and transportation including well-maintained in-country biorepositories for storage. Analysis of these tissue samples enable patient stratification, prognostic assessments via biomarker developments, and pharmacologic studies that would be impractical to perform by acquiring large numbers of trial participants. Few biobanks are emerging, and these include H3A biorepositories in Uganda, Stellenbosch, and Cape Town, and 54 Gene and ARGO biobanks in Nigeria. Start-up costs for establishing a biobank is cost intensive but may be cost saving if centralized, given that longer-term maintenance costs also need to be factored in. More of such should be established and should be supported by all researchers in sub-Saharan Africa.

5. The provision of funds for pilot studies:

Before launching a big trial, preliminary feasibility assessment or trials is encouraged to build local research culture. In this regard, ARGO introduced grants to fund research initiative of indigenous early career researchers in 2015. Since its introduction, six researchers have benefited from this opportunity.

6. Secure protected time for African researchers:

Clinical and basic researchers in Africa have no guaranteed protected time as they are expected to be involved in clinical and teaching assignments in addition to administrative duties. There should be increased advocacy to policymakers, opinion leaders, and mentors on possible innovative ways to ensure protected time for researchers.

7. Strengthen health authority and government agency clinical trial approval processes:

Different countries in Africa have well laid-out sets of standard regulatory and institutional review board (IRB) guidelines for conducting a clinical trial.^39^ Puppalwar et al^39^ reviewed some of the guidelines for some of the African countries.^39^ Good understanding of the guidelines can help to reduce bottleneck encountered in getting the appropriate approvals.

In conclusion, Africa needs to conduct more clinical trials to reverse the dismal statistic of hosting < 2% of global clinical trials despite being host to 16.7% of the population and 25% of the global burden of disease. All stakeholders must work together to ensure this is a reality. The economies of African countries are growing with a population of 1.2 billion, which is expected to double in 2050. As such, there are commercial and economic opportunities for investors in addition to a rising middle and younger population with disposable income and growing awareness of health care.
